# Editorial: Artificial Intelligence and Machine Learning Applications in Plant Genomics and Genetics

**DOI:** 10.3389/frai.2022.959470

**Published:** 2022-06-27

**Authors:** Aalt D. J. van Dijk, Shin-Han Shiu, Dick de Ridder

**Affiliations:** ^1^Bioinformatics Group, Wageningen University, Wageningen, Netherlands; ^2^Department of Plant Biology, Michigan State University, East Lansing, MI, United States; ^3^Department of Computational Mathematics, Science, and Engineering, Michigan State University, East Lansing, MI, United States

**Keywords:** machine learning, plant science, artificial intelligence, genomics, genetics

In plants, as in other species, genotypic variation in combination with environmental variation leads to differences in the biochemical makeup of cells, measurable as molecular phenotypes. These in turn influence physiological and developmental traits such as organ formation and plant growth, and eventually traits relevant in agriculture, such as yield and stress tolerance. Thus, relating genotypes to phenotypes yields fundamental insights into the regulation of important processes in plant development and physiology, but also provides the ability to predict yield and quality traits in specific environments, which is essential in basic plant science and molecular breeding of resilient plants in changing environments.

Recent technological developments have revolutionized measurements on plant genotypes and phenotypes, leading to routine production of large, complex data sets. A resulting challenge in both fundamental and applied plant sciences is to use this genetic, genomic and phenomic data to predict or even explain phenotypes from the underlying genotypes in different environments. The potential of artificial intelligence and machine learning methods (AI/ML) to extract relevant information out of raw phenotype measurement data, such as images and video, is already widely recognized. However, the subsequent analysis of phenotypes measured at different scales or the linking of these phenotypes to genotypes increasingly calls for processing and integration of large, noisy, and heterogeneous data sets. To exploit the full potential of these data, AI/ML are now starting to be widely applied in many areas in plant science and plant breeding. Next to applications of existing methods, novel methodologies are being developed for challenges specific to this area (e.g., comparative and evolutionary analyses of wide varieties of complex genomes, reconstruction of molecular networks) and specific applications in plant breeding, such as genomic prediction and selection.

This collection brings together a number of interesting studies at the interface of AI/ML and plant sciences. In fundamental genomics research, rapidly accumulating omics data provides useful features for predicting a wide range of labels such as molecular activities (e.g., gene expression, molecular interactions), physiological function (e.g., enzyme or signaling pathways), and phenotypes (e.g., morphological measurements, flowering time). The first paper in this collection, by Cho et al., is an example of such an application, where genomic and protein sequences were used to predict gene expression in specific plant tissues with high accuracy. This was accomplished by using Markov model classifiers to turn sequence information into feature vectors, which served as input to classical algorithms to predict tissue-specific expression.

In another paper, Yoosefzadeh-Najafabadi et al. study the use of hyperspectral measurement data in soybean, which is predictive of yield and can be measured easily in early growth stages. They take a two-step approach, where yield is predicted based on hyperspectral bands (phenotype-phenotype prediction), and next hyperspectral bands are related to genotypes using a genome-wide association study (GWAS) approach (genotype-phenotype). They compare conventional statistical methods to a support vector regression method, which automatically selects relevant features and find that the latter provides better insight in the biology underlying the relation between hyperspectral bands and yield.

Finally, two of the papers in this collection deal with genomic prediction (GP), which aims to predict (complex) traits using genomic data. Well established quantitative genetics approaches exist for GP, but ML may offer advantages as it imposes less strong assumptions (Gaussianity, linearity etc.). So far, however, ML approaches have not shown consistent improvement in prediction performance; hence, development of ML for GP is an active field of research. One key issue in any ML application is how to encode the input data, and this is addressed from different angles by both papers on GP in this issue. Gabur et al. investigated how feature selection could benefit GP. Their results indicate that using a reduced set of markers, prediction accuracy can be improved, which is relevant for cost-efficient use of genotype data. Galli et al. compare two alternative approaches to enable deep learning on genomic markers, using either the genetic relatedness matrix, or converting markers to “genomic images,” which are then used as input. Although prediction performance was highly conditioned on specifics of the data (e.g., which trait was predicted), under certain conditions the genomic images based method showed the best performance.

The papers in this collection clearly demonstrate the opportunities provided by AI/ML in plant phenomics, genomics and genetics. However, it is also clear that various challenges are still ahead. As explained in more detail in a recent review on the use of ML in plant sciences and plant breeding (van Dijk et al., 2021), the success of ML critically depends on the availability of sufficiently large numbers of samples that have reasonable quality, are representative of the population and that share a sufficient number of common features. Moreover, compared to more traditional statistical methods, for various applications a disadvantage of ML is that estimating confidence in the predictions is less straightforward, and that model interpretation is not always feasible. Methodology to deal with these issues is currently under active development. Note that model interpretation by dissecting AI/ML models is useful for revealing the underlying biological mechanisms, and is also important for revealing quality issues and potential biases in the data. An additional, important consideration is that new ML methodologies are needed for problems with complex, heterogeneous and/or variable data which current methods find difficult to handle. As described above, data integration and feature representation are covered in some of the work in this collection. Beyond the examples here, a wide variety of data at various scales exists, ranging from the molecular level (e.g., single cell transcriptomics) to the landscape scale (e.g., satellite based weather conditions at the field level). Efficient ways to encode such data, which take relevant structure in the data into account, will need to be further developed and offer great opportunities for novel discoveries.

## Author Contributions

All authors listed have made a substantial, direct, and intellectual contribution to the work and approved it for publication.

## Funding

This work was partially supported by the U.S. Department of Energy Great Lakes Bioenergy Research Center (BER DE-SC0018409) and the U.S. National Science Foundation (DEG-1655386, DGE-1828149, and IOS-2107215) to S-HS.

## Conflict of Interest

The authors declare that the research was conducted in the absence of any commercial or financial relationships that could be construed as a potential conflict of interest.

## Publisher's Note

All claims expressed in this article are solely those of the authors and do not necessarily represent those of their affiliated organizations, or those of the publisher, the editors and the reviewers. Any product that may be evaluated in this article, or claim that may be made by its manufacturer, is not guaranteed or endorsed by the publisher.
